# Individual and Obstetric Risk Factors of Preeclampsia among Singleton Pregnancy in Hospitals of Southern Ethiopia

**DOI:** 10.1155/2021/7430827

**Published:** 2021-01-20

**Authors:** Kassahun Fikadu Tessema, Feleke Gebremeskel, Firdawek Getahun, Nega Chufamo, Direslgne Misker

**Affiliations:** ^1^Department of Midwifery, Arbaminch University, Arab Minch, Ethiopia; ^2^School of Public Health, Arbaminch University, Arab Minch, Ethiopia; ^3^Department of Obstetrics and Gynecology, Arbaminch University, Arab Minch, Ethiopia

## Abstract

**Background:**

Preeclampsia is the second most common problem that causes maternal morbidity and complication in low-income countries. In contrast to death due to other direct causes, preeclampsia-related death is appeared to be connected with multiple factors; yet, factors have paucity and are limited. Considering the clinical significance, this study aimed to identify that individual and obstetric factors of preeclampsia can be an input for disease identification involving clinicians in southern Ethiopia.

**Methods:**

A case-control study was conducted among mothers with a singleton pregnancy who attended perinatal care in all six public hospitals in the provinces around the Omo stream. A sample size of 487 women with a singleton pregnancy (163 cases and 326 controls) was involved in the study. All cases were enrolled, while controls were selected consecutively using a random sampling technique. Data were gathered using a structured questionnaire and data extraction sheet. Descriptive data were presented using percentages and numbers. Multivariable logistic regression analysis was carried out to identify factors at a *p* value of less than 0.05.

**Results:**

There was a statistically significant association between the family history of hypertension (AOR = 2.42, 95% CI: 1.16–5.05), no pregnancy interval (AOR = 1.62; 95% CI: 1.03–2.55), and normal body mass index (AOR = 0.42, 95% CI: 0.21–0.87) and the occurrence of preeclampsia.

**Conclusion:**

Primary relatives with a history of chronic hypertension and no pregnancy interval were identified as risk factors of preeclampsia, while having a normal body mass index was found to be a protective factor of preeclampsia occurrence. To improve early detection and timely management of preeclampsia, the clinician should give attention to women who have no previous childbirth and whose close relatives had a history of chronic hypertension, as well as working on the protective factor is recommended.

## 1. Background

Both in the high income and low-resource setting countries across the globe, hypertensive disorders of pregnancy have persisted as a global burden that attributes to maternal and perinatal morbidity and mortality [1]. Hypertensive disorders of pregnancy are appeared to be one of the top five causes of maternal morbidity and mortality [2]. According to the recent World Health Organisation (WHO) report, more than 9% of maternal death in Africa was due to hypertensive disorders of pregnancy [1, 3]. About 9% of maternal death in Ghana was also because of gestational hypertension. A five-year retrospective study conducted in Shoa district revealed that approximately 12% of maternal death happened because of hypertensive disorder of pregnancy, while about 17% of maternal death in Ethiopia was due to pregnancy-induced hypertension [4, 5].

The incidence of preeclampsia in developing countries is estimated to be seven times higher than the developed world [6]. The global incidence of preeclampsia is estimated to be from 5% to 14%, while in developing countries, the incidence is increased by 4% [7]. The overall prevalence of preeclampsia across studies varies from 4% to 23% [8]. In the USA, preeclampsia affects 4% of pregnancies [9], in Kenya (6.1%) [10], Nigeria (2%–14%) [6], and Ethiopia (4.74%) [11]. Regarding the severe form of the disease, severe preeclampsia accounts 25% of all preeclampsia cases [7].

The severe form of preeclampsia results in maternal system dysfunction, and its consequences involve maternal and fetal morbidity and death. Worldwide, nearly 12% of direct maternal death is contributed by the complications of preeclampsia [12]. In Ethiopia, preeclampsia contributes 10% of all maternal mortality and 16% of direct maternal death [13]. Unlike maternal death due to haemorrhage/obstructed labour or sepsis, maternal death due to preeclampsia has been increasing and remains a major public health problem. Nevertheless, in the majority of cases, maternal death due to preeclampsia is preventable using an effective and timely provision of care [14].

Giving due attention to this public health problem may contribute to the overall effort of reducing maternal mortality in Ethiopia. Ethiopia has identified preeclampsia as one of the contributions of maternal death, and the government has been working on quality improvement aspects of the maternal health services. The intervention involves the job training for clinicians, which aimed to enable them to detect and manage preeclampsia using magnesium sulphate, as well as supportive supervision and mentoring and availability of supplies to health facilities with the support of health development partners [15]. However, the unpredictable nature of the disease and limited evidence on factors of preeclampsia made the effort tiresome. Considering the benefit of documentation on factors of preeclampsia in reducing the overall incidence of preeclampsia, this study was aimed to determine individual and obstetric factors of preeclampsia in hospitals of southern Ethiopia.

## 2. Methods

### 2.1. Study Area and Period

This case-control study design was conducted in six district public health hospitals in southern Ethiopia. All the districts are located along with the Omo river stream. In the Gamo, Goffa, Derashe, and Konso districts are in the upstream, while South Omo district is regarded as the low stream. The study was carried out at the hospital of each district, where each has one government owing hospital during the study period between February and August 2018. The hospitals included in this study were Arbaminch General Hospital, Konso District Hospital, Sawla District Hospital, Chencha District Hospital, Jinka General Hospital, and Gidole District Hospital. Cases were pregnant women with a new onset of elevated diastolic blood pressure of 90 mmHg and/or systolic blood pressure of 140 mmHg on two consecutive records of four–six hours apart or highly elevated blood pressure (diastolic blood pressure ≥110 mmHg and/or a systolic blood pressure ≥160 mmHg) of a single occurrence, plus protein in the urine estimated to be 300 gm per day or urine dipstick record of >1+ after 20 weeks of gestation, and controls were pregnant women not diagnosed with preeclampsia [1, 16]. Gestational age was calculated referring to the pregnant women's recall of the last normal menstrual period (LMP). A documented early ultrasound record was used to compute the gestational age for those who failed to remember the exact date of their last menstrual period. To prevent misclassification, pregnant women who did not remember LMP and had no ultrasound record were excluded.

### 2.2. Sample Size Calculation and Sampling Technique

Epi-Info version 7.2 was used to compute the estimated sample size using double population proportion formula by assuming antenatal counselling as a factor for with lowest odds ratio of 1.8 and 40.2% cases among controls from previously published research studies [17]. In addition, 95% confidence interval, 5% marginal error, and 80% power were considered. The calculated sample size was 443, and after adding a 10% nonresponse rate, the final sample size was 487 (163 cases and 326 controls).

All the six hospitals were selected purposely because they are public health hospitals that cover the majority of the maternal health service utilization in the catchment. Consecutively appearing cases for antenatal and delivery services at these hospitals during the study period were enrolled. The selection of cases was carried out after the duty physician (general practitioner (GP) or gynaecologist) had the diagnosis preeclampsia. Thus, for each selected case, two consecutively appearing pregnant women who had fulfilled the criteria for controls were enrolled. This ensured a case and control ratio of 1 : 2. This was made using the delivery register as the sampling frame list. The sampling frame was updated based on the antenatal attendees and deliveries that take place in the study hospitals. The sample size distributed between the six hospitals was proportional to their antenatal and delivery caseload. Participants were informed to sign a consent form, and for those subjects below 18 years, parent/guardian was required to sign an assent form on a separate sheet.

### 2.3. Data Collection Instrument and Personnel

Data were gathered using a structured and pretested data questionnaire by face-to-face interview. To ensure consistency, the English version data collection tool was translated into Amharic (the national working language), and it was translated back to English by using language experts. The questionnaire consists of three parts, such as sociodemographic factors, personnel, and obstetric-related variables. It was prepared by referring to different works of literature [18–22]. Twelve Amharic speaking diploma and degree level midwives were recruited as data collectors. Six medical doctors supervised the data collection process in coordination with the principal investigator. Supervisors had a daily check on the completeness, consistency, and clarity of the data before the start of data collection in the following day. Along with the investigator, the supervisor had informed data collectors to review the questionnaire for completeness and any missing variables once before the discharge of the participant. To assure data quality, data collectors and supervisors were taken two days training on reviewing technique, the objective of the study, and ethical aspects involving informed consent and confidentiality issues. Moreover, the pretest of the data collection was carried out in area outside of the study area (in Wolaita Sodo Comprehensive and Teaching Specialized Hospital) on 10% (50) of samples.

### 2.4. Measurement

To avoid the white coat effect and transient elevation of blood pressure because of anxiety and exhaustion, we allowed the participant to take rest for five to ten minutes before the start of blood pressure. To ensure the functionality of the cuff, data collectors checked on a daily basis. A mercury sphygmomanometer was used, and the apparatus covers the upper two-thirds arm. At the antenatal clinic, the record of blood pressure was performed while the women were seated in the upright position. The record of blood pressure for women in the delivery unit left lateral position was used. At both service delivery units, right brachial pressure was taken. During the procedure, the cuff was inflated at a rate of 2-3  mmHg per second. Systolic blood pressure (SBP) was noted as the first Korotkoff sound has been heard, and diastolic blood pressure (DBP) was obtained when the fourth Korotkoff sound was heard. Upon detection of abnormal or unsatisfactory measurements, the measurement was repeated at least four hours apart to confirm the diagnosis. However, the final confirmation was done by the duty physician. Those pregnant women with the diagnosis of severe preeclampsia (blood pressure of 160/110 mmHg at any record) were sent for immediate medical management and reevaluation. Information about proteinuria and other relevant data were accessed from maternity charts.

### 2.5. Data Management, Analysis, and Interpretation

After manual checking of the filled questionnaire for completeness, cleaning and data entry was made into Epi-Info version 7.2 statistical software and transferred to Statistical Package for Social Sciences (SPSS) version 26.0 for further analysis. For all variables, data accuracy and missed values were checked with a frequency run, while cross-tabulation and the chi-square test were made to compare cases and controls for categorical variables. Frequency (%) was used to describe categorical variables. Odds ratio (OR) and 95% confidence intervals were calculated to measure relevant variables in bivariable regression analysis and variables with *p* value less than or equal to 0.25, as well as evidence from previously published research outputs and plausibility were considered to fit variables into the final multivariable regression analysis model. The predictive value of the model was assessed using Hosmer–Lemeshow goodness-of-fit test statistics. In multivariable regression analysis, adjusted odds ratio (AOR) and the corresponding 95% confidence intervals for cases were calculated to see the strength of the association between the outcome and explanatory variables. Finally, independent variables with a *p* value of less than 0.05 were considered as a statistically significant factor of preeclampsia.

## 3. Results

### 3.1. Sociodemographic Characteristics of the Study Participants

From a total of 487 (163 case and 326 controls) participants, 7 cases had withdrawn from the interview. A total of 480 (156 (32.0%) cases and 326 (67.9%) controls) who came for perinatal care in Omo district hospitals participated and completed the interview; this gives a response rate of 98.56% for both cases and controls. The mean ages of the study subjects in both groups were 25.6 with a SD of ±5.5. The majority of 99 (63.5%) cases were between 20 and 29 years of age. The majority of 208 (63.8%) of the control groups fell between 20 and 29 years of age. More than 151 (96.8%) cases and 303 (92.9%) controls were married. Regarding educational status, the majority of 43 (27.6%) cases and 97 (29.8%) controls had attended primary education. Eighty-seven (55.6%) cases were housewives compared to 168 (51.5%) controls, on which they had a similar duty in their house (Table 1).

Thirty-seven (23.7%) cases had a family history of chronic hypertension. Regarding the medical illness of participants, the family history of the diabetes mellitus was 6.4% in cases and 9.2% in controls. The personnel history of diabetes mellitus was also reported as 5.8% in cases and 4% in controls. The history of modern contraceptives use was slightly lower in cases than does in controls, in which it was reported to be 46.9% in cases compared to 49.1% in control counterparts. Regarding the body mass index of participants, 92 (59.0%) cases had a normal body mass index (Table 2).

### 3.2. Obstetric-Related Factors of Participants

Regarding the number of pregnancies, 60 (38.5%) cases and 133 (40.8%) controls had more than one child. Sixty-five (41.7%) of the cases and 166 (50.9%) of the controls had conceived their pregnancy between two to five years interval (Table 3).

Multivariable analysis revealed that having no child, normal BMI, and a family history of chronic hypertension was found to have a significant statistical association with preeclampsia.

Concerning the body mass index, it was revealed that having normal weight index was found to protect the women from preeclampsia. The odds of developing preeclampsia were decreased by 58% for women with normal weight compared to their underweight counterparts (AOR = 0.42, 95% CI: 0.21–0.87). In this study, having no child was independently associated with preeclampsia. The odds of developing preeclampsia were 1.62 times higher in women who had no pregnancy interval compared to women with an interpregnancy interval between two and five years (AOR = 1.62; 95% CI: 1.03–2.55). The multivariable analysis also revealed that the risk of developing preeclampsia was 2.4 times higher among women who had close relatives with a history of chronic hypertension (AOR = 2.42, 95%CI: 1.16–5.05) (Table 4).

## 4. Discussion

A hospital-based case-control study was conducted with the aim of identifying individual and obstetric factors of preeclampsia. After the multivariable analysis has been adjusted for a singleton pregnancy, it found no pregnancy interval and having primary relatives with a history of chronic hypertension as a risk factor, while normal body mass index was a protective factor for preeclampsia. The novel finding in this study was that a weak but significant association exists between normal BMI and preeclampsia in a singleton pregnancy where it would be a marker of nutritional deprivation.

The findings of this study are supported by several studies [23–27]. Levels of physical activity among subjects might also suggest the occurrence of elevated blood pressure in pregnant women, in which the risk of high blood pressure among subjects with high physical activity and normal weight was reduced nearly by half, as a prospective cohort study revealed [23]. Another study also reported that there was a weak but significant negative association between BMI and blood pressure. This might suggest a link between malnutrition and elevated BP [25]. A study from Italy reported that participants with normal weight had a lower prevalence of elevated blood pressure compared with enrollees with overweight [26]. Furthermore, a recent review comparing the US and non-US population indicated that a normal body mass index (BMI) was correlated with a low incidence of hypertension [27]. A multicountry study in Africa and Asia countries including Ethiopia revealed that BMI has a negative or weak association with blood pressure. The weak but significant linear relationship between BMI and maternal blood pressure in Ethiopia was limited to those women whose BMI includes normal BMI. In this regard, undernutrition (42.7%) and the physical activity level may explain the negative association between normal body mass index and increased blood pressure [24]. Eventhough we found a negative association between normal BMI and preeclampsia, undernutrition may be an important factor of elevated blood pressure in the area where it is prevalent. Thus, careful evaluation of the contribution of undernutrition to preeclampsia would be important.

According to this study, women who had no child were independently associated with preeclampsia. This finding is in line with several studies [28–30]. It has been shown that the incidence of preeclampsia was three times higher among women who had no child [28]. Another study conducted in Norwegian women indicated that the odds of developing preeclampsia were increased by 3.6 times among women with no previous childbirth history [31]. Maternal immunity maladaptation is the suggested explanation linked to nulliparity, in which how the maternal immune system reacts to early trophoblastic cells invasion leads to maladaptation of the spiral arterioles [8]. The extent of immunological exposure is the reason for an increased or decreased risk of preeclampsia in subsequent pregnancies [32].

Concerning to the medical history of participants, the family history of chronic hypertension with primary relatives was a statistically significant factor that increases the risk of preeclampsia. This finding is consistent with studies conducted in Dessie [22], Addis Ababa [33], Gedio [34], India and Kenya [17, 35], and Pakistan [36]. This might be because of a behavioural or genetic-related factor that contributes to the physiologic susceptibility of preeclampsia.

### 4.1. Limitations of the Study

Using a case-control study design and enrollment of a representative sample is the strength of this study. Due to the retrospective nature of the study, recall bias can be the challenge. The facility-based study limits the finding to be limited to women attending perinatal care in a hospital. Despite a wide range of geographic area, interested researchers may consider as a basis for future work.

## 5. Conclusions

In light of the findings, it can be concluded that having primary relatives who had a history of chronic hypertension and no interpregnancy interval were independent risk factors, whereas having normal body mass index protects women from developing preeclampsia. Therefore, it is recommended that planning the maternal health program at the district level should incorporate preventive and control strategies focusing on the risk factors and women should be advised to keep the normal bodyweight index with regular activity and adequate nutrition.

## 6. Declarations

We, the undersigned, agree to accept responsibility for the scientific, ethical, and technical conduct of the research project mentioned and for the provision of required progress reports and financial settlements as per the terms and conditions of the university if the grant is awarded as the result of this application.

## Figures and Tables

**Table 1:** Sociodemographic characteristics of women enrolled for a case-control study conducted among singleton pregnant women attending perinatal care in Omo district hospitals, southern Ethiopia. Individual and family-related characteristics of participants.

Variable	Outcome variable		X^2^	Crude odds ratio (95% CI)	*p* value
	Preeclampsia, *n* (%)	Controls, *n* (%)			
Participant age					
<20 years	22 (14.1%)	36 (11.0%)	1.56	1 (ref)	
20–29	99 (63.5%)	208 (63.8%)		0.78 (0.44–1.39)	0.40
30–34	24 (15.4%)	51 (15.6%)		0.77 (0.38–1.58)	0.48
35 and above	11 (7.1%)	31 (9.5%)		0.58 (0.24–1.38)	0.22

Residence					
Urban	81 (51.9%)	178 (54.6%)	0.30	0.90 (0.61–1.32)	0.58
Rural	75 (48.1%)	148 (45.4%)		Ref	

Women's occupation					
Housewife	87 (55.6%)	168 (51.5%)	3.08	1.41 (0.57–3.47)	0.46
Merchant	23 (14.7%)	47 (14.4%)		1.33 (0.49–3.61)	0.58
Government employee	15 (9.6%)	43 (13.2%)		0.95 (0.33–2.70)	0.92
Private worker	8 (5.1%)	23 (7.1%)		0.94 (0.28–3.08)	0.92
Students	16 (10.3%)	26 (8.0%)		1.67 (0.58–4.86)	0.35
Others	7 (4.5%)	19 (5.8%)		Ref	

Husband's occupation					
Government employee	45 (28.8%)	92 (28.2%)	6.58	0.71 (0.31–1.66)	0.43
Private employee	37 (23.7%)	103 (31.6%)		0.52 (0.22–1.23)	0.14
Farmer	55 (35.3%)	89 (27.3%)		0.90 (0.39–2.08)	0.80
Merchant	8 (5.1%)	26 (8.0%		0.45 (0.15–1.24)	0.15
Others	11 (7.1%)	16 (4.9%)		Ref	

Marital status					
Unmarried	5 (3.2%)	23 (7.2%)	2.59	Ref	
Married	151 (96.8%)	303 (92.9%)		2.29 (0.86–6.06)	0.09

Educational level					
No formal education	40 (25.6%)	71 (21.8%)	2.48		
Primary level	43 (27.6%)	97 (29.8%)		0.79 (0.46–1.33)	0.37
Secondary level	37 (23.7%)	94 (28.8%)		0.70 (0.41–1.20)	0.20
College and above	36 (23.1%)	64 (19.6%)		0.99 (0.57–1.75)	0.99

**Table 2:** Individual and family-related factors of women enrolled for a case-control study conducted among singleton pregnant women attending perinatal care in Omo district hospitals, southern Ethiopia.

Variable	Outcome variable		*X* ^2^	Crude odds ratio (95% CI)	*p* value
	Preeclampsia, *n* (%)	Controls, *n* (%)			
Family history of chronic hypertension					
No apparent history	119 (76.3%)	276 (84.7%)	5.73	1 (ref)	
Primary relatives only	18 (11.5%)	20 (6.1%)		2.09 (1.07–4.09)	0.03
Secondary relatives only	19 (12.2%)	30 (9.2%)		1.47 (0.79–2.71)	0.22

Family history of diabetes mellitus					
Yes	10 (6.4%)	30 (9.2%)	1.08	0.68 (0.32–1.42)	
No	146 (93.6%)	296 (90.8%)		Ref	0.30

Personnel history of diabetes mellitus					
Yes	9 (5.8%)	13 (4.0%)	0.77	1.47 (0.62–3.53)	
No	147 (94.2%)	313 (96.0%)		Ref	0.38

Maternal asthma					
Yes	11 (92.9%)	14 (4.3%)	1.63	1.69 (0.75–3.82)	0.21
No	145 (7.1%)	312 (95.7%)		Ref	

History of abortion					
Yes	27 (17.3%)	60 (18.4%)	0.09	0.92 (0.56–1.53)	0.77
No	129 (82.7%)	266 (81.6%)		Ref	

Haematologic status					
Low haemoglobin	56 (35.9%)	106 (32.5%)	0.54	1.16 (0.78–1.74)	0.46
Normal haemoglobin	100 (64.1%)	220 (67.5%)		Ref	

Contraceptive use					
No contraceptive use	83 (53.2%)	166 (50.9%)	1.83	Ref	
Injectable	50 (32.1%)	98 (30.1)		1.02 (0.66–1.57)	0.93
Implants	14 (9.0%)	43 (13.2%)		0.65 (0.34–1.26)	0.20
Oral pills	9 (5.8%)	19 (5.8%)		0.95 (0.41–2.19)	0.90

Body mass index (BMI)					
Underweight	18 (11.5%)	19 (5.8%)	8.12	Ref	
Normal	92 (59.0%)	215 (66.0%)		0.45 (0.23–0.90	0.02
Overweight	39 (25.0%)	86 (26.4%)		0.48 (0.23–1.01)	0.05
Obese	7 (4.5%)	6 (1.8%)		1.23 (0.35–4.37)	0.75

**Table 3:** Obstetric-related characteristics of women enrolled for a case-control study on factors of preeclampsia among pregnant women in Omo district hospitals, southern Ethiopia. The identified factors of preeclampsia.

Variable	Outcome variable		*X* ^2^	Crude odds ratio (95% CI)	*p* value
	Preeclampsia, *n* (%)	Controls, *n* (%)			
Fetal sex					
Male	87 (55.8%)	177 (54.3%)	0.10	1.06 (0.72–1.56)	0.76
Female	69 (44.2%)	149 (45.7%)		Ref	

Parity					
Nulliparous	58 (37.2%)	100 (30.7%)	2.53	1.84 (0.69–4.86)	0.22
Primiparous	32 (20.5%)	74 (22.7%)		1.37 (0.50–3.75)	0.54
Multiparous	60 (38.5%)	133 (40.8%)		1.43 (0.54–3.76)	0.47
Grandmultiparous	6 (3.8%)	19 (5.8%)		Ref	

Pregnancy interval					
No prior childbirth	59 (37.8%)	107 (32.8%)	3.73	1.41 (0.92–2.16)	0.12
Less than 2 years	32 (20.5%)	53 (16.3%)		1.54 (0.91–2.60)	0.11
Two to five years	65 (41.7%)	166 (50.9%)		Ref	

Pregnancy from new partner					
Yes	33 (21.2%)	63 (19.3%)	0.22	1.12 (0.70–1.80)	0.64
No	123 (78.8%)	263 (80.7%)		Ref	

ANC follow-up status					
Yes	106 (67.9%)	212 (65.0%)	0.40	1.14 (0.76–1.71)	
No	50 (32.1%)	114 (35.0%)		Ref	0.53

Nutritional counselling					
Yes	138 (88.5%)	304 (93.3%)	3.2	0.56 (0.29–1.07)	0.07
No	18 (11.5%)	22 (6.7%)		Ref	

**Table 4:** Multivariable output for a case-control study on personnel and obstetric factors of preeclampsia among pregnant women attending hospitals in southern Ethiopia.

Variable	Outcome variable		Adjusted odds ratio (95% CI)	*p* value
	Preeclampsia, *n* (%)	Controls, *n* (%)		
Husband's occupation				
Government employee	45 (28.8%)	92 (28.2%)	0.64 (0.27–1.55)	0.34
Private employee	37 (23.7%)	103 (31.6%)	0.46 (0.19–1.12)	0.09
Farmer	55 (35.3%)	89 (27.3%)	0.78 (0.33–1.87)	0.58
Merchant	8 (5.1%)	26 (8.0%)	0.32 (0.10–1.02)	0.06
Others	11 (7.1%)	16 (4.9%)	Ref	
Body mass index (BMI)				
Underweight	18 (11.5%)	19 (5.8%)	Ref	
Normal weight	92 (59.0%)	215 (66.0%)	0.42 (0.21–0.87)	0.02
Overweight	39 (25.0%)	86 (26.4%)	0.49 (0.23–1.06)	0.07
Obese	7 (4.5%)	6 (1.8%)	1.46 (0.39–5.55)	0.58
Pregnancy interval				
No prior childbirth	59 (37.8%)	107 (32.8%)	1.62 (1.03–2.55)	0.04
Less than 2 years	32 (20.5%)	53 (16.3%)	1.74 (1.00–3.03)	0.05
Two to five years	65 (41.7%)	166 (50.9%)	(1.01–3.03)	
Marital status				
Unmarried	5 (3.2%)	23 (7.2%)	Ref	
Married	151 (96.8%)	303 (92.9%)	2.58 (0.93–7.17)	0.07
Family history of diabetes mellitus				
Yes	10 (6.4%)	30 (9.2%)	2.31 (0.91–5.84)	0.08
No	146 (93.6)	296 (90.8)	Ref	
Family history of chronic hypertension				
No history	119 (76.3%)	276 (84.7%)	Ref	
Primary relatives	18 (11.5%)	20 (6.1%)	2.42 (1.16–5.05)	0.02
Secondary relatives	19 (12.2%)	30 (9.2%)	1.32 (0.68–2.55)	0.42
Personnel history of diabetes mellitus				
Yes	9 (5.8%)	13 (4.0%)	0.35 (0.12–1.01)	0.05
No	147 (94.2%)	313 (96.0%)	Ref	

## Data Availability

Datasets used in this study are available from the corresponding author upon reasonable request.

## Abbreviations

ANC: Antenatal care
AOR: Adjusted odds ratio
BMI: Body mass index
COD: Crude odds ratio
CI: Confidence interval
WHO: World Health Organisation.

## References

[1] Steegers E. A., Von Dadelszen P., Duvekot J. J., Pijnenborg R. (2010). Pre-eclampsia. *The Lancet*.

[2] Osungbade K. O., Ige O. K. (2011). Public health perspectives of preeclampsia in developing countries: implication for health system strengthening. *Journal of Pregnancy*.

[3] Say L., Chou D., Gemmill A. (2014). Global causes of maternal death: a WHO systematic analysis. *The Lancet Global Health*.

[4] Mekonnen W., Gebremariam A. (2018). Causes of maternal death in Ethiopia between 1990 and 2016: a systematic review with meta-analysis. *Ethiopian Journal of Health Development*.

[5] Garomssa H., Dwivedi A. (2008). Maternal mortality in Ambo Hospital: a five-year retrospective review. *Ethiopian Journal of Reproductive Health*.

[6] Osungbade K. O. I. O. (2011). Public health perspectives of preeclampsia in developing countries: implication for health system strengthening. *Journal of Pregnancy*.

[7] Lamminpää R. V.-J. K., Gissler M., Heinonen S. (2012). Preeclampsia complicated by advanced maternal age: a registry-based study on primiparous women in Finland 1997–2008. *BMC Pregnancy Childbirth*.

[8] Kahnamouei-Adam F. A. F., Hamidimoghaddam S. (2015). Prevalence of pre-eclampsia and eclampsia risk factors among pregnant women, 2011-2013. *International Journal of Advances in Medicine*.

[9] Bibbins-Domingo K., Grossman D. C., Curry S. J. (2017). Screening for preeclampsia: US preventive services task force recommendation statement. *JAMA*.

[10] NO A. (2012). *Factors Contributing to Adverse Outcomes of Pre-eclampsia Among Pregnant Women Attending Antenatal Clinics in Kibera Slums*.

[11] Tesfa E. N. E., Gizaw S. T., Zenebe Y. (2020). Prevalence and determinants of hypertensive disorders of pregnancy in Ethiopia: a systematic review and meta-analysis. *PloS One*.

[12] Filippi V. C. D., Ronsmans C., Graham W., Say L. (2016). *Levels and Causes of Maternal Mortality and Morbidity*.

[13] Maternal A. A. (2010). Mortality trend in Ethiopia. *Ethiopian Journal of Health Development*.

[14] World Health Organization (2011). *WHO Recommendations for the Prevention and Treatment of Pre-eclampsia and Eclampsia*.

[15] Currie S., De Graft-Johnson J., Galloway R., Sheehan C., Smith J. (2012). *Interventions for Impact on Essential Obstetric and Newborn Care*.

[16] (2011). A conceptual framework for the revision of the ICD-10 classification of mental and behavioural disorders. *World Psychiatry*.

[17] Logan G. G., Njoroge P. K., Nyabola L. O., Mweu M. M. (2020). Determinants of preeclampsia and eclampsia among women delivering in county hospitals in Nairobi, Kenya. *F1000Research*.

[18] Skursky N., Sedlander E., Barboza K. (2015). Abstracts from the 38th annual meeting of the society of general internal medicine. *Journal of General Internal Medicine*.

[19] Sutapa Agrawal G. K. W. (2014). Prevalence and risk factors for pre-eclampsia in Indian women: a national cross sectional study. *Journal of Women Health Issues and Care*.

[20] Tebeu P. M., Foumane P., Mbu R., Fosso G., Biyaga P. T., Fomulu J. N. (2011). Risk factors for hypertensive disorders in pregnancy: a report from the maroua regional hospital, Cameroon. *Journal of Reproduction & Infertility*.

[21] Yogev Y., Nir M., Bardin R. (2010). Pregnancy outcome at extremely advanced maternal age. *American Journal of Obstetrics and Gynecology*.

[22] Tessema G. A., Tekeste A., Tadesse A. A. (2015). Preeclampsia and associated factors among pregnant women attending antenatal care in Dessie referral hospital, Northeast Ethiopia: a hospital-based study. *BMC Pregnancy and Childbirth*.

[23] Hu G., Barengo N. C., Tuomilehto J., Lakka T. A., Nissinen A., Jousilahti P. (2004). Relationship of physical activity and body mass index to the risk of hypertension: a prospective study in Finland. *Hypertension*.

[24] Tesfaye F., Nawi N. G., Van Minh H. (2007). Association between body mass index and blood pressure across three populations in Africa and Asia. *Journal of Human Hypertension*.

[25] Salahudeen A. K., Fleischmann E. H., Bower J. D., Hall J. E. (2004). Underweight rather than overweight is associated with higher prevalence of hypertension: BP vs BMI in haemodialysis population. *Nephrology Dialysis Transplantation*.

[26] Landi F., Calvani R., Picca A. (2018). Body mass index is strongly associated with hypertension: results from the Longevity Check-up 7+ Study. *Nutrients*.

[27] Younus A., Aneni E. C., Spatz E. S. (2016). A systematic review of the prevalence and outcomes of ideal cardiovascular health in US and non-US populations. *Mayo Clinic Proceedings*.

[28] Luo Z.-C., An N., Xu H.-R., Larante A., Audibert F., Fraser W. D. (2007). The effects and mechanisms of primiparity on the risk of pre-eclampsia: a systematic review. *Paediatric and Perinatal Epidemiology*.

[29] Wandabwa J., Doyle P., Kiondo P., Campbell O., Maconichie N., Welishe G. (2010). Risk factors for severe pre-eclampsia and eclampsia in Mulago Hospital, Kampala, Uganda. *East African Medical Journal*.

[30] Cormick G., Betrán A. P., Ciapponi A., Hall D. R., Hofmeyr G. J. (2016). Inter-pregnancy interval and risk of recurrent pre-eclampsia: systematic review and meta-analysis. *Reproductive Health*.

[31] Ødegård R. A., Vatten L. J., Nilsen S. T., Salvesen K. A., Austgulen R. (2000). Risk factors and clinical manifestations of pre-eclampsia. *BJOG: An International Journal of Obstetrics and Gynaecology*.

[32] Li D.-K., Wi S. (2000). Changing paternity and the risk of preeclampsia/eclampsia in the subsequent pregnancy. *American Journal of Epidemiology*.

[33] Grum T., Seifu A., Abay M., Angesom T., Tsegay L. (2017). Determinants of pre-eclampsia/Eclampsia among women attending delivery Services in Selected Public Hospitals of Addis Ababa, Ethiopia: a case-control study. *BMC Pregnancy and Childbirth*.

[34] Mareg M., Molla A., Dires S., Berhanu Mamo Z., Hagos B. (2020). Determinants of preeclampsia among pregnant mothers attending antenatal care (ANC) and delivery service in gedeo zone, southern Ethiopia: case control-study. *International Journal of Women’s Health*.

[35] Ganesh K. S., Unnikrishnan B., Nagaraj K., Jayaram S. (2010). Determinants of pre-eclampsia: a case-control study in a district hospital in South India. *Indian Journal of Community Medicine: Official Publication of Indian Association of Preventive & Social Medicine*.

[36] Shamsi U., Hatcher J., Shamsi A., Zuberi N., Qadri Z., Saleem S. (2010). A multicentre matched case-control study of risk factors for preeclampsia in healthy women in Pakistan. *BMC Women’s Health*.

